# Case Report: Synchronous Removal and Implantation of Peritoneal Dialysis Catheter Using Bilateral Transversus Abdominis Plane Block

**DOI:** 10.3389/fmed.2022.828930

**Published:** 2022-03-01

**Authors:** Ante Jakšić, Božidar Vujičić, Diana Deša, Antun Gršković, Ivan Vukelić, Josip Španjol, Sanjin Rački, Dean Markić

**Affiliations:** ^1^Department of Urology, University Hospital Rijeka, Rijeka, Croatia; ^2^Department of Nephrology, Dialysis and Kidney Transplantation, University Hospital Rijeka, Rijeka, Croatia; ^3^Faculty of Medicine, University of Rijeka, Rijeka, Croatia; ^4^Department of Anesthesiology and Intensive Care, University Hospital Rijeka, Rijeka, Croatia

**Keywords:** case-reports, end-stage renal disease, peritoneal dialysis catheter, regional anesthesia, transversus abdominis plane (TAP) block

## Abstract

**Background:**

Peritoneal dialysis (PD) surgery include PD catheter insertion and removal. Both procedures require the use of anesthesia. The end-stage renal disease (ESRD) patients usually have severe comorbidities. The general anesthesia, because of its negative systemic effect, should be omitted in this vulnerable group of the patients. Transversus abdominis plane (TAP) block as a newer method of regional anesthesia is a technique without systemic effect and recently started to be used in ESRD patients for PD catheter placement and/or removal. Here we report a patient in whom we for the first time simultaneously removed and implanted a PD catheter by using a bilateral transversus abdominis plane block.

**Case Presentation:**

The patient was an 80-year-old man who was admitted for removal of malfunctioned PD catheter. Since the patient opted for staying on PD simultaneous implantation of catheter was planned. Because of his age and significant comorbidities, general anesthesia was avoided and bilateral TAP block become our option. In the same anesthesia, using bilateral TAP block, the old PD catheter was removed and a new one was implanted. Until now the patient is on regular PD without any complications.

**Conclusion:**

The TAP block could be used as a primary anesthetic technique in ESRD patients for PD surgery even for synchronous removal and implantation of PD catheter.

## Introduction

Peritoneal dialysis (PD) is a method of renal replacement therapy (1). End-stage renal disease (ESRD) patients often have severe cardiovascular, respiratory, gastrointestinal, hematologic, and skeletal comorbidities resulting in significantly higher mortality rates among hemodialysis and PD patients (2).

PD catheter implantation and removal are surgical procedures that require adequate anesthesia. General anesthesia is commonly used anesthetic technique for the insertion and removal of a PD catheter. However, general anesthesia can have significant adverse effects on the cardiovascular and respiratory systems and newer anesthesia techniques could be used for this purpose. One of these methods is transversus abdominis plane (TAP) block as a part of a regional anesthesia technique. It is a peripheral nerve block targeting nerves situated in the fascial layer between the transversus abdominis and internal oblique muscles (3). Application of local anesthetic in this plane caused anesthesia of the anterolateral abdominal wall which is used for insertion and/or removal of PD catheter. Regional anesthesia has negligible systemic effects, and recently the use of the TAP block was described in PD catheter procedures (4–9). Herein, for the first time, we report a synchronous removal and insertion of PD catheter using TAP block.

## Case Report

An 80-year-old patient with ESRD was admitted to our department for synchronous removal and implantation of a PD catheter. The body weight of the patient was 98 kg, height 185 cm and his BMI was 28.6 kg/m^2^. The patient had a long history of arterial hypertension, type 2 diabetes mellitus, mitral and tricuspid regurgitation with secondary cardiomyopathy. In 2017 a PD catheter using TAP block, as the primary anesthetic procedure, was inserted.

However, 3 years later, the patient developed bacterial peritonitis caused by Streptococcus species, which was successfully treated with a first-generation cephalosporin for 14 days. The development of bacterial peritonitis caused the malfunction of the PD catheter (inflow obstruction) with inadequate dialysis exchange. The patient was informed about the possibility of other therapeutic options and opted to continue treatment with PD. The patient was preoperatively assessed by anesthesiologist and his functional capacity using metabolic equivalent of task (MET) was defined as poor (below 4) because his main activity includes only eating, dressing, toileting, walking indoors, and light housework. He was unable to walk 2 blocks on level ground without stopping due to symptoms. Since he has significant comorbidities his physical status was classified using American Society of Anesthesiologists Physical Status Classification System (ASA) as ASA III (patient with severe systemic disease). Considering the patient's comorbidities, general condition, age, and possible complications of general anesthesia, we decided to perform synchronous removal and placement of the PD catheter under regional anesthesia.

**Figure 1:**
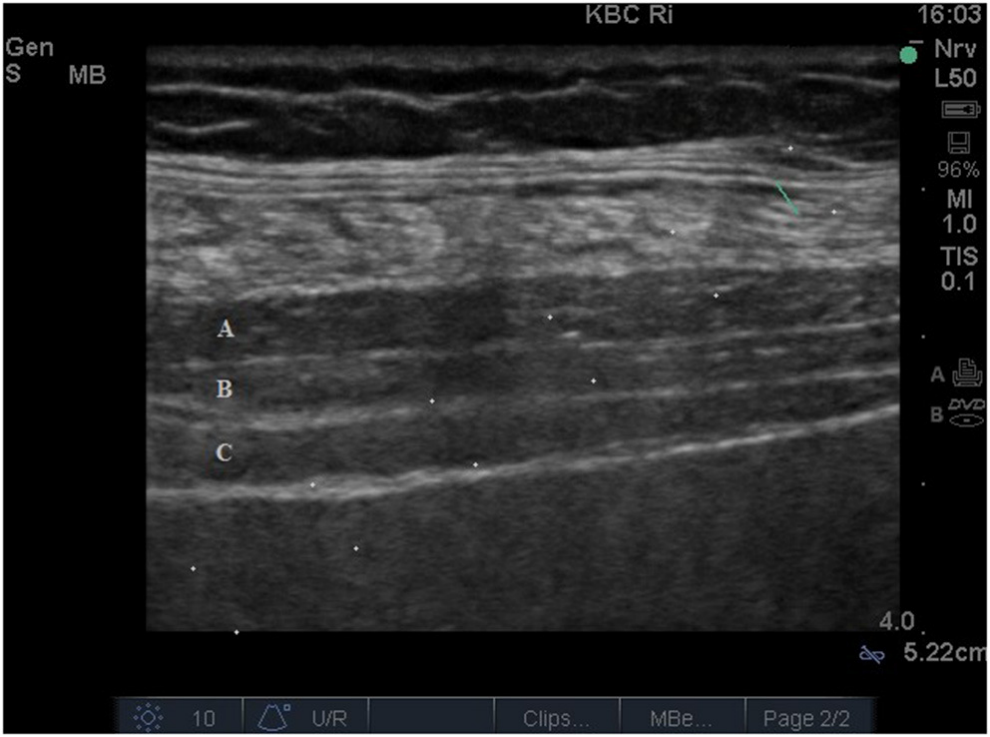
Ultrasound image showing all three muscles of the abdominal wall: **(A)** external oblique, **(B)** internal oblique and **(C)** transversus abdominis muscle.

**Figure 2:**
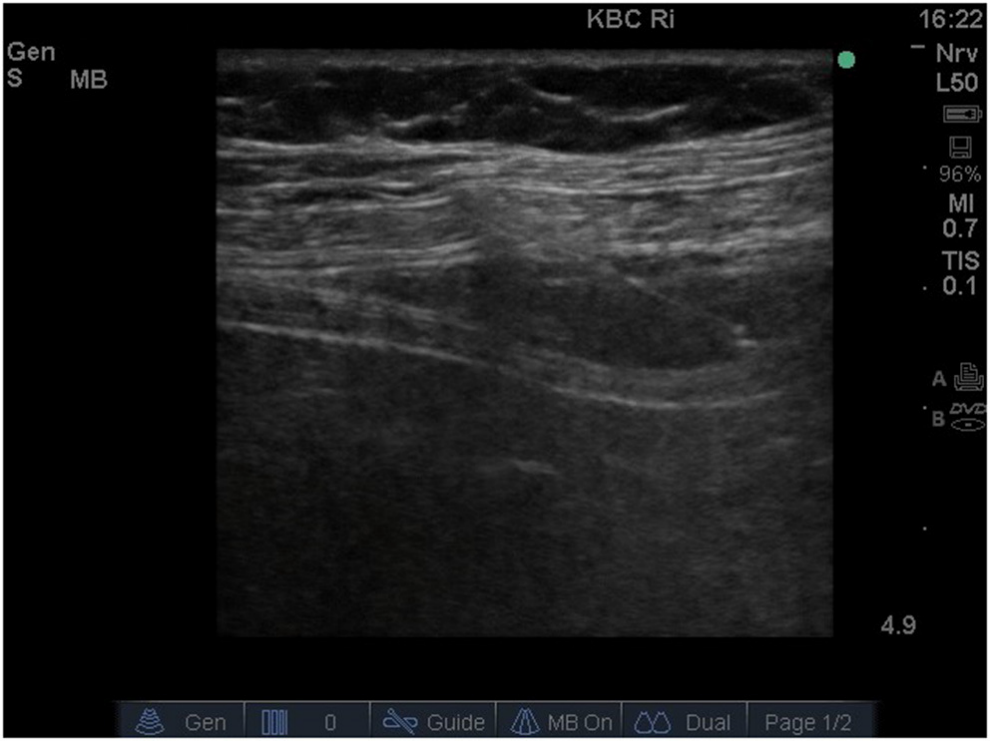
Ultrasound image demonstrating a needle placed in the space between the internal oblique and transversus abdominis muscles (transversus abdominis plane) with the injection of local anesthetic into the target area.

The transversus abdominis field was identified and approached with a combined ultrasound-guided subcostal and posterior approach, as we previously described (5). Briefly, after identification of the correct plane, 30 ml of 0.25% levobupivacaine hydrochloride per side was injected between the transversus abdominis muscle and the internal oblique muscle under the ultrasound guidance (Figures 1, 2). We tested the adequacy of the TAP block with the cold sensation test and pinprick test. About 30 mins after injecting the anesthetic, excision of the skin scar from the previous implantation on the right side of the abdomen was performed, the PD catheter was found and removed. Then vertical paramedian infraumbilical incision (minilaparotomy) was made on the left side and a straight, 42 cm long Tenckhoff catheter with double-cuff was implanted. During surgery, the patient received sufentanil (10 μg) and midazolam 3 mg for better analgesic/sedative effect. In addition, 10 ml of 1% xylocaine was administrated subcutaneously on both sides during surgery for better analgesia. The operative time, measured from a skin incision on the right side to skin closure on the left side, was 40 mins. The patient classified surgery procedure complete painless and felt just little discomfort after surgery without need for painkillers.

On the first postoperative day, we started automated PD and after 1 month we continued with the continuous ambulatory peritoneal dialysis, which is still used today.

## Discussion

Regional anesthesia become popular in many fields of medicine because of its good anesthetic effect with negligible systemic effect. Primary, TAP block was used for control of postoperative pain after abdominal surgeries as adjunct to the general anesthesia (10–12). In very isolated cases this procedure was used as the primary anesthetic technique in the elderly patients with significant comorbidities which has increased risk for general anesthesia (13). Recently, the use of TAP block, as a primary anesthetic technique, for PD catheter surgery was a next step in the implementation of this procedure in medical practice.

The first author to describe the successful TAP block procedure for PD catheter placement was Varadarajan in 60/73 (82%) patient (4). In this study, two regional anesthetic techniques were used, TAP block and rectus sheath block (4). However, our group described the successful use of a unilateral TAP block as the sole anesthetic technique in 55/60 (91.7%) adult patients for PD catheter insertion (7). Other studies have also described the successful implantation of PD catheter using TAP block (6, 8). Chatterjee et al. report a success rate for PD catheter implantation of 94.2% (6).

Yamamoto et al. (9) were the first to describe their experience with PD catheter removal using TAP block in three patients. Our previous study showed successful use of TAP block for PD catheter removal in 13/14 patients (92.9%), with only one patient requiring general anesthesia (7). There are several reports on the use of bilateral TAP block instead of general anesthesia in high-risk patients with multiple comorbidities, but none for PD catheter surgery (13, 14).

Local anesthesia (LA) is another frequently used technique for PD catheter placement. LA could be combined with intravenous sedation for better efficacy (15). However, the local infiltration of an anesthetic can cause edema and bleeding at the incision site, which disturbs the surgical field. For most patients, especially obese ones, local infiltration of anesthetic must be repeated. These repeated injections can induce fear and anxiety in patients (5).

Some concerns could be made because of use of total amount of 60 ml of 0.25% levobupivacaine (75 mg per side, total dose of 150 mg) and 20 ml of 1% xylocaine (total dose of 200 mg). Since TAP block was bilateral the dosage of applied anesthetic was higher than usually but still in the safety zone. The maximal recommended dose for levobupivacaine is 2 mg/kg or 200 mg (we used total of 150 mg) (16). For the lidocaine maximal recommended dose is 5 mg/kg or 350 mg (200 mg in our patient) (17). So, administered anesthetic dosage was in the safety zone and potential negative systemic effects are not expected.

In this patient we added local anesthesia for better analgesia because of two incisions more than usually. We have two main incisions (right paramedian incision for the extraction and left paramedian incision for the implantation pf the PD catheter) and two minor lateral incisions (for the exit site of the extracted PD catheter—right side and implanted PD catheter-left side). Usually, when we made unilateral implantation or removal of PD catheter the local anesthetic was not needed. Only, in very rare circumstances, when the patient's report pain or discomfort we added local anesthetic. In this case because the surgery was a more extensive than usually, we decide to added local anesthetic. A total dose of used lidocaine for one side was lower (10 ml) compared to 20–40 ml when PD catheter is implanted using local anesthesia with sedation (15, 18). For better analgosedation, we used fentanyl in a low dose and midazolam which was enough for our procedure.

Based on our previous experience with regional anesthesia, we used a bilateral combined (posterior + lateral) TAP block as the primary anesthetic technique. To the best of our knowledge, this is the first case describing the use of a bilateral combined TAP block during surgery for synchronous removal and PD catheter implantation.

## Conclusion

The use of newer anesthetic techniques may provide a safer alternative to general anesthesia for PD surgery. The TAP block could be used as primary anesthetic technique in ESRD patients for synchronous removal and implantation of PD catheter.

## Data Availability Statement

The raw data supporting the conclusions of this article will be made available by the authors, without undue reservation.

## Ethics Statement

Ethical review and approval was not required for the study on human participants in accordance with the local legislation and institutional requirements. The patients/participants provided their written informed consent to participate in this study. Written informed consent was obtained from the individual(s) for the publication of any potentially identifiable images or data included in this article.

## Author Contributions

DM, AJ, DD, BV, IV, JŠ, SR, and AG researched literature and planned treatment protocol. AG, IV, DM, AJ, and DD were involved in operative treatment. DM, BV, SR, and JŠ were involved in postoperative follow-up. DM and BV obtained informed consent. AJ wrote the first draft of the manuscript. All authors reviewed and edited the manuscript, and approved the final version.

## Conflict of Interest

The authors declare that the research was conducted in the absence of any commercial or financial relationships that could be construed as a potential conflict of interest.

## Publisher's Note

All claims expressed in this article are solely those of the authors and do not necessarily represent those of their affiliated organizations, or those of the publisher, the editors and the reviewers. Any product that may be evaluated in this article, or claim that may be made by its manufacturer, is not guaranteed or endorsed by the publisher.

## References

[1] National Kidney Foundation. Kidney Disease Quality Outcomes Initiative (K/DOQI). (2021). Available online at: http://www.kidney.org/professionals/kdoqi/guidelines.cfm (accessed November 18, 2021).

[2] Goodkin DA Bragg-Gresham JL Koenig KG Wolfe RA Akiba T Andreucci VE. Association of comorbid conditions and mortality in hemodialysis patients in Europe, Japan, and the Unites States: the Dialysis Outcomes and Practice Patterns Study (DOPPS). J Am Soc Nephrol. (2003) 14:3270–7. 10.1097/01.asn.0000100127.54107.57 14638926

[3] Webster K. The transversus abdominis plane (TAP) block: abdominal plane regional anesthesia. Anesthesia. (2008) 24:24–9.

[4] Varadarajan Y Balasubramaniyam R. Ultrasound guided rectus sheath and transversus abdominis plane block (TAP) for continuous ambulatory peritoneal dialysis (CAPD) catheterization—our experience. Nephrol Dial Transplant. (2012) 27:ii464A.

[5] Markić D Vujičić B Ivanovski M Krpina K Gršković A Živčić-Cosić S. Peritoneal dialysis catheter placement using an ultrasound-guided transversus abdominis plane block. Blood Purif. (2015) 39:274–80. 10.1159/000381005 25925151

[6] Chatterjee S Bain J Christopher S Gopal TV Raju KP Mathur P. Role of regional anesthesia for placement of peritoneal dialysis catheter under ultrasound guidance: our experience with 52 end-stage renal disease patients. Saudi J Anaest. (2015) 9:132–5. 10.4103/1658-354X.152838 25829899 PMC4374216

[7] Markić D Vujičić B Ivanovski M Krpina K Gršković A Rahelić D. Peritoneal dialysis catheter surgery using transversus abdominis plane block. Perit Dial Int. (2017) 37:429–33. 10.3747/pdi.2016.00198 28408712

[8] Hecquet E Bonamy C Levesque C Bechade C Ficheux M Lobbedez T. Peritoneal dialysis catheter insertion under TAP block procedure: a pilot study. Nephrol Ther. (2015) 11:164–8. 10.1016/j.nephro.2015.01.005 25921734

[9] Yamamoto H Shido A Sakura S Saito Y. Monitored anesthesia care based on ultrasound-guided subcostal transversus abdominis plane block for continuous ambulatory peritoneal dialysis catheter surgery: case series. J Anesth. (2016) 30:156–60. 10.1007/s00540-015-2074-0 26337833

[10] McDonnell JG Curley G Carney J Benton A Costello J Maharaj CH. The analgesic efficacy of transversus abdominis plane block after cesarean delivery: a randomized controlled trial. Anesth Analg. (2008) 106:186–91. 10.1213/01.ane.0000290294.64090.f3 18165577

[11] McDonnell JG O'Donnell B Curley G Heffernan A Power C Laffey JG. The analgesic efficacy of transversus abdominis plane block after abdominal surgery: a prospective randomized controlled trial. Anesth Analg. (2007) 104:193–7. 10.1213/01.ane.0000250223.49963.0f 17179269

[12] Freir NM Murphy C Mugawar M Linnane A Cunningham AJ. Transversus abdominis plane block for analgesia in renal transplantation: a randomized controlled trial. Anesth Analg. (2012) 115:953–7. 10.1213/ANE.0b013e3182642117 22763899

[13] Vuong JT McQuillan PM Messaris E Adhikary SD. Transversus abdominis plane block as the primary anesthetic for laparotomy. J Anaesthesiol Clin Pharmacol. (2014) 30:419–21. 10.4103/0970-9185.137284 25190958 PMC4152690

[14] Mishra L Pani N Mishra D Patel N. Bilateral transversus abdominis plane block as a sole anesthetic technique in emergency surgery for perforated peritonitis in a high risk patient. J Anaesthesiol Clin Pharmacol. (2013) 29:540–2. 10.4103/0970-9185.119140 24249994 PMC3819851

[15] Jabbour E Fütterer C Zach S Kälsch AI Keese M Rahbari NN. Implantation of a peritoneal dialysis catheter in patients with ESRD using local anesthesia and remifentanil. PLoS One. (2021) 16:e0259351. 10.1371/journal.pone.0259351 34735524 PMC8568152

[16] Cox B Durieux ME Marcus MA. Toxicity of local anaesthetics. Best Pract Res Clin Anaesthesiol. (2003) 17:111–36. 10.1053/bean.2003.0275 12751552

[17] El-Boghdadly K Pawa A Chin KJ. Local anesthetic systemic toxicity: current perspectives. Local Reg Anesth. (2018) 11:35–44. 10.2147/LRA.S154512 30122981 PMC6087022

[18] Henderson S Brown E Levy J. Safety and efficacy of percutaneous insertion of peritoneal dialysis catheters under sedation and local anaesthetic. Nephrol Dial Transplant. (2009) 24:3499–504. 10.1093/ndt/gfp312 19556299

